# Age-Related Patterns in Health Literacy and Health Information–Seeking Behavior in a Korean Population: Cross-Sectional Study

**DOI:** 10.2196/86992

**Published:** 2026-09-23

**Authors:** Da Hae Kwon, Young Dae Kwon

**Affiliations:** 1 Incheon Public Health Policy Institute, Incheon, Republic of Korea; 2 Department of Humanities and Social Medicine, College of Medicine, The Catholic University of Korea, 222 Banpo-Daero, Seocho-gu, Seoul, 06591, Republic of Korea, 82 2-3147-8303, 82 2-3147-8480; 3 Catholic Institute for Public Health and Healthcare Management, The Catholic University of Korea, Seoul, Republic of Korea

**Keywords:** health information–seeking behavior, health literacy, age differences, logistic models, Korea, internet use

## Abstract

**Background:**

Health information–seeking behavior (HISB) is a critical component of personal health management. Health literacy (HL) shapes whether and how people seek health information, but its role across age groups remains underexplored. Korea’s highly digitalized information environment and rapidly aging population provide an important context for examining age-group differences in HL and HISB.

**Objective:**

This study examined age-group differences in the association of HL with 2 related stages of HISB—HISB experience and, conditional on engagement, source-based HISB type—and assessed whether the roles of HL, education, and health-related factors differed across age groups.

**Methods:**

Cross-sectional data from the 2020‐2021 Korea Health Panel and 2021 HL Supplementary Survey (N=7910) were analyzed. HL was measured using the European Health Literacy Survey Questionnaire-16-item (HLS-EU-Q16). HISB type was classified as source-based “active seeking” or “passive acquisition” according to the respondents’ top-ranked information source. Age-stratified multivariable logistic regression was used for HISB experience and type, and sensitivity analyses examined age×HL interactions and potential selection into the HISB type sample.

**Results:**

Mean HL scores were lower in older age groups (14.10, SD 2.94 in ages 19‐44 years vs 8.34, SD 4.65 in ≥65 years). HISB prevalence was highest in the youngest group (1072/1608, 66.7%) and lowest in the oldest group (1052/3487, 30.2%). Among those who engaged in HISB, 92.7% (993/1072) of the youngest group and 48.2% (507/1052) of the oldest group were classified in the active-seeking category. Primary age-stratified and interaction analyses showed that HL was significantly associated with HISB experience and type among adults aged 45‐64 and ≥65 years, but not among those aged 19‐44 years, whereas education and health-related factors were more salient among younger adults. The HL-HISB type association was sensitive to modeling selection.

**Conclusions:**

Age-group patterns were evident in HISB experience and source-based type. HL was a more prominent correlate among middle-aged and older adults, while education and health-related factors were more salient among younger adults. Age-tailored strategies that also consider structural, motivational, and digital-access factors alongside HL are needed.

## Introduction

### Background

Health information–seeking behavior (HISB) is a fundamental component of personal health management and a recognized social determinant of health. In an increasingly digital environment, individuals are expected to engage proactively with diverse sources to make informed decisions on prevention, diagnosis, and treatment [1-3]. Health information seeking has been linked to increased health knowledge, improved decision-making, healthier behaviors, and favorable health outcomes [1-8].

Health literacy (HL) is critical in shaping individuals’ HISB [1,5]. HL affects whether individuals seek information, how actively they engage with it, and whether they can evaluate and apply what they find [6,8]. Those with higher HL tend to use more diverse and credible information sources, demonstrate greater initiative in information seeking, and report higher confidence in managing their health [9,10]. Conversely, individuals with low HL may encounter barriers in navigating health systems, communicating with providers, or even recognizing the need for additional information [7,11-13].

Among multiple dimensions of HISB, the type of behavior, particularly the distinction between active information seeking and passive information acquisition, has been used as an important analytical lens [5,7,8]. At the conceptual level, active seeking refers to purposeful, goal-directed efforts to find health information, whereas passive acquisition refers to incidental exposure without deliberate information seeking [5,7,14]. This distinction is analytically useful because active and passive forms of information engagement may differ in their associations with health-related outcomes. HL is thus considered a predictor of whether people seek health information and how they do so [8].

Age-group differences in HL and HISB have been widely documented: older adults generally have lower HL, which may reflect educational, cognitive, and technological factors [13-16], and, despite having greater health needs, are less likely to actively seek information [17-19]. Although typically higher in HL, younger adults may rely on less vetted sources or exhibit superficial engagement with information [20]. These contrasting patterns underscore the importance of examining age-specific dynamics in the HL-HISB relationship. They can be interpreted through the lens of information-seeking frameworks such as the Comprehensive Model of Information Seeking (CMIS), which emphasizes that information seeking is shaped by individual characteristics, perceived health-related relevance, and information-source factors [21]. This perspective is useful for understanding why HL, education, and perceived health needs may play different roles in active versus passive HISB across age groups [21,22].

### Objectives

Previous studies have primarily focused on HL or HISB, either separately or within specific subgroups [23,24]. Few have analyzed their relationship using nationwide adult panel data [15,25,26], and even fewer have explored age-specific differences, despite clear theoretical and empirical reasons to do so [27]. Building on the distinction between active information seeking and passive information acquisition [5,7], this study addresses this gap by examining not only whether HL is associated with HISB, but also whether the correlates of HISB type differ across age groups. We focus on whether HL, educational attainment, and perceived health needs are differentially associated with active versus passive HISB across age groups. The South Korean context, characterized by rapid population aging alongside a highly digitalized health information environment, provides a relevant setting for examining these age-specific dynamics. Using data derived from a nationally designed panel, we investigate (1) how HISB patterns vary by age group, (2) whether HL is associated with an individual’s likelihood of engaging in HISB, and (3) whether the source-based HISB type (active vs passive) varies according to HL within each age group.

## Methods

### Data

This cross-sectional study used data from the 2020‐2021 Korea Health Panel (KHP) and 2021 HL Supplementary Survey, which were collected within a nationally designed panel framework. The KHP, jointly administered by the Korea Institute for Health and Social Affairs and the National Health Insurance Service, is an annual longitudinal survey that assesses patterns of health care utilization, expenditures, and key determinants, including individual health conditions and socioeconomic characteristics. The sampling frame was derived from the 2016 Korean Population and Housing Census for the second panel cohort, introduced in 2020, to ensure broad national coverage.

The KHP data structure combines cross-sectional and longitudinal elements, with repeated annual measurements collected via face-to-face interviews and self-reported questionnaires. As a longitudinal panel dataset, the KHP can capture both between-individual variation at specific time points and within-individual changes over time. However, for this study, only between-individual variation at a single time point was examined. A single cross-sectional wave was used to align with the HL survey period and to assess age-group differences in HL and health behavior.

The HL Supplementary Survey was conducted between March and July 2021 among respondents who had completed the KHP. This supplemental module included a validated and standardized tool for assessing HL among respondents from nationally designed KHP. Approximately 9530 adults aged 19 years or older completed the HL module. To ensure completeness and consistency, 1620 individuals were excluded due to missing responses on key variables, including health care utilization (n=1482), private health insurance status (n=95), high-risk drinking (n=38), smoking status (n=4), and type of health coverage (n=1). The final analytic sample consisted of 7910 adults. A flow diagram of the sample selection process is shown in Figure S1 in Multimedia Appendix 1.

### Operational Definitions of HISB Experience and Type

To assess HISB in greater detail, participants were first asked whether they had sought any health-related information during the past 12 months. This binary (yes or no) variable defined the overall presence of HISB. Among those who responded affirmatively, the type of HISB was categorized as either active seeking or passive acquisition based solely on the respondent’s top-ranked information source from a predefined list of 9 options. We treated this source as the respondent’s primary health information source. Drawing on prior literature, we classified the sources by the mode of access typically associated with each source [3,14,26,28-31]. Information sources such as health care professionals, government websites, hospital websites, internet portal searches, and targeted searches on platforms like YouTube (Google) were assigned to the active-seeking category [3,14,28,29]. In contrast, sources such as television, radio, newspapers, magazines, books, and interpersonal channels such as family or friends were classified under the passive acquisition category [26,29-31]. The classification scheme used to determine HISB type is summarized in Table 1. The KHP HL Supplement identified respondents’ primary health information source but did not assess whether the reported source was intentionally accessed or merely encountered incidentally. Accordingly, this classification should be interpreted as a source-based proxy for active seeking and passive acquisition rather than a direct measure of behavioral intentionality. For example, some sources, including YouTube, newspapers and magazines, and interpersonal channels, may involve either active searching or passive exposure depending on the context of use.

**Table 1.** Classification of health information sources into health information–seeking behavior (HISB) types among Korean adults: a cross-sectional analysis of the 2020‐2021 Korea Health Panel and 2021 Health Literacy Supplementary Survey^a^.

Type	Sources
Active information–seeking category	Health care professionals (doctors, nurses, pharmacists, etc) [29] Government public sector channels (website, YouTube, and SNS^b^) [3,14] Hospital or public health center channels (website, YouTube, and SNS) [3,14] Internet portals [14] YouTube (various sources) [28]
Passive information acquisition category	Television [26,29,31] Radio [26,29] Newspapers, magazines, or books (print and online) [29] Family, friends, colleagues, and acquaintances [29,30]

a HISB type was classified based on the respondent’s top-ranked information source and represents a source-based proxy rather than a direct measure of intentionality.

b SNS: social network service.

### Variables and Measurements

HL was measured using the European Health Literacy Survey Questionnaire-16-item (HLS-EU-Q16), a validated instrument adapted for the Korean population. This tool comprises 16 items covering health care, disease prevention, and health promotion domains. Respondents were asked to rate each item based on perceived difficulty using a four-point Likert scale: “very difficult,” “difficult,” “easy,” and “very easy,” with an additional “do not know” option. In this study, responses were dichotomized into “easy” (1 point) and “difficult” (0 points), while “do not know” responses were treated as missing. The total score ranged from 0 to 16, with higher scores indicating greater HL. The overall HL score was considered missing if a respondent had any missing values. For descriptive purposes, HL scores were categorized as inadequate (0‐8), problematic (9-12), or adequate (13-16) [32], but in multivariable analyses, HL was used as a continuous variable.

A range of covariates was selected based on prior research linking them to HL and HISB and included to account for potential confounding. Sociodemographic variables comprised gender, age group, marital status, educational attainment, household income, employment status, type of health coverage, and private health insurance enrollment [26,33-35]. Age was categorized into three groups: 19‐44 years (young adults), 45‐64 years (middle-aged adults), and 65 years and older (older adults). Given the cross-sectional design, these age groups were interpreted as reflecting age-cohort differences rather than pure physiological or cognitive aging effects, because chronological age and birth cohort could not be disentangled in the current data [36,37]. Educational attainment was grouped into elementary school or less, middle or high school, and college or above. Household income was equivalized by dividing total monthly household income by the square root of the number of household members. Employment status was categorized as economically active or inactive. Health coverage included National Health Insurance and Medical Aid. Health behavior variables included current smoking, high-risk drinking, and walking [25,33,34]. Current smoking was defined as the use of conventional cigarettes or nicotine-containing electronic cigarettes at the time of the survey. High-risk drinking was defined as consuming 7 or more drinks per occasion for men (5 or more for women) at least twice a week during the prior year. Walking behavior was defined as walking for 30 minutes or more at least 5 days per week. Health status and health care utilization variables included self-rated health (SRH; on a five-point Likert scale), number of chronic diseases (categorized as none, one, or two or more), health-related quality of life (assessed using the EQ-5D index), disability status, number of outpatient visits, hospitalization experience, and whether the respondent had a usual source of care [15,26,33,35]. Unless otherwise specified, all covariates reflected values as of December 31, 2020.

### Statistical Analysis

Descriptive statistics summarized participants’ demographic, behavioral, and health-related characteristics. Chi-square tests were used to examine the distribution of key variables across age groups, including health information–seeking experience and behavior type. The KHP provides survey weights for the full-panel cohort, but none were calibrated specifically for the restricted analytic sample of respondents who completed the 2021 HL Supplementary Survey and had complete data on all analytic variables. Applying the full-panel weights to this complete-case subsample could yield weighted estimates that do not accurately represent either the original target population or the final analytic sample. We therefore retained unweighted analyses as the primary approach.

Primary inferential analyses distinguished two related stages of HISB: whether respondents reported any HISB experience, and, among those who did, the source-based type of HISB. This reflected the outcome data structure, in which HISB experience was observed for the full analytic sample, whereas HISB type was defined only for respondents who reported HISB experience. First, multivariable logistic regression was used to examine the association between HL and the likelihood of seeking health information in the past 12 months (yes or no). Second, among respondents who had engaged in information seeking, an additional multivariable logistic regression model was used to assess the association between HL and the source-based HISB type (active seeking vs passive acquisition). These stage-specific models constituted the primary analyses because they addressed complementary inferential objectives: HISB engagement in the full analytic sample and HISB type conditional on engagement. For each stage, models were estimated for the relevant overall sample and separately within each age group (19‐44, 45‐64, and ≥65 years).

To examine whether the association between HL and each outcome differed across age groups, two pooled multivariable logistic regression models incorporating age×HL interaction terms were estimated in a separate sensitivity analysis: one for HISB experience in the full analytic sample and one for HISB type among respondents who had engaged in HISB. The overall significance of the age×HL interaction terms was evaluated using joint Wald *χ*² tests, and age-specific associations between HL and each outcome were estimated from the pooled interaction models. Separately, potential selection into the HISB type analytic sample was evaluated using a separate Heckman-type 2-step sensitivity analysis. Covariate coding for this sensitivity analysis was harmonized with that used in the primary HISB type model. In the first step, a probit model was used to estimate the probability of selection into the HISB type analytic sample. In the second step, the inverse Mills ratio (IMR) derived from the first-step probit model was included in a logistic regression model comparing active seeking with passive acquisition. Although the original Heckman model was developed for continuous second-stage outcomes, related extensions have been applied to binary second-stage outcomes [38-40]. All analyses were conducted using Statistical Analysis System (SAS) version 9.4 (SAS Institute Inc.), and statistical significance was determined using a two-tailed *P* value threshold of .05.

### Ethical Considerations

The study was conducted in accordance with the principles outlined in the Declaration of Helsinki. The study was approved by the Institutional Review Board of the Catholic University of Korea (MC24ZASI0109), and informed consent was waived because publicly available secondary data were used. To ensure privacy and confidentiality, all data were fully anonymized and deidentified prior to access. Participant compensation was not applicable because this study involved a secondary analysis of survey data.

## Results

### Participant Characteristics

Among the 7910 participants, the largest proportion (3487/7910, 44.1%) was aged 65 years and older, followed by 35.6% (2815/7910) aged 45‐64 years and 20.3% (1608/7910) aged 19‐44 years. Education level differed significantly by age, with nearly half (1734/3487, 49.7%) of the oldest group having completed elementary school or below, compared with just 0.3% (4/1608) in the youngest group. Age differences were also observed in private health insurance enrollment rates, which were approximately 90% (4015/4423) among participants aged 19‐64 years but dropped to 53.1% (1852/3487) among those aged 65 years or older. A similar pattern appeared in economic activity: 68.1% (1095/1608) of individuals aged 19‐44 years were economically active, compared to 42.4% (1479/3487) of the oldest group. Health behaviors and SRH also varied with age. The oldest adults reported the highest rate of walking (1697/3487, 48.7%) and the lowest prevalence of current smoking (334/3487, 9.6%) and high-risk drinking (142/3487, 4.1%). Older age groups reported poorer SRH, with a lower proportion reporting good health and a higher proportion with multiple chronic conditions.

### HISB Prevalence and Type

HISB also showed notable age-group differences. While 66.7% (1072/1608) of adults aged 19‐44 years had sought health information, only 30.2% (1052/3487) of those aged 65 years and older had done so. Furthermore, among those who had sought information, 51.8% (545/1052) of the oldest group was classified in the passive acquisition category, compared with only 7.3% (78/1072) of the youngest group. The overall mean HL score was 10.96 (SD 4.79), and mean scores were lower in older age groups (Table 2).

**Table 2.** General characteristics by age group among Korean adults: a cross-sectional analysis of the 2020‐2021 Korea Health Panel and 2021 Health Literacy Supplementary Survey.

Variable	Age group (years)						Chi-square or *F* test (*df*)	Chi-square or *F* test (*df*)
	All (N=7910)		19‐44 (n=1608)		45‐64 (n=2815)			
Sex, n (%)							2.33 (2)^a^	2.33 (2)^a^
Male	3369 (42.6)		658 (40.9)		1208 (42.9)			
Female	4541 (57.4)		950 (59.1)		1607 (57.1)			
Marital status, n (%)							2004.23 (4)^a,b^	2004.23 (4)^a,b^
Married	5715 (72.3)		1004 (62.4)		2277 (80.9)			
Divorced, separated, or widowed	1481 (18.7)		45 (2.8)		415 (14.7)			
Not married	714 (9.0)		559 (34.8)		123 (4.4)			
Education level, n (%)							3508.29 (4)^a,b^	3508.29 (4)^a,b^
Elementary school or below	2001 (25.3)		4 (0.3)		263 (9.3)			
Middle or high school	3442 (43.5)		356 (22.1)		1618 (57.5)			
College or above	2467 (31.2)		1248 (77.6)		934 (33.2)			
Health care coverage, n (%)							49.29 (2)^a,b^	49.29 (2)^a,b^
National Health Insurance	7561 (95.6)		1581 (98.3)		2701 (96.0)			
Medical Aid	349 (4.4)		27 (1.7)		114 (4.1)			
Private health insurance, n (%)							1447.63 (2)^a,b^	1447.63 (2)^a,b^
Yes	5867 (74.2)		1487 (92.5)		2528 (89.8)			
No	2043 (25.8)		121 (7.5)		287 (10.2)			
Economic activity, n (%)							576.82 (2)^a,b^	576.82 (2)^a,b^
Yes	4543 (57.4)		1095 (68.1)		1969 (70.0)			
No	3367 (42.6)		513 (31.9)		846 (30.1)			
Current smoking, n (%)							121.34 (2)^a,b^	121.34 (2)^a,b^
Yes	1146 (14.5)		294 (18.3)		518 (18.4)			
No	6764 (85.5)		1314 (81.7)		2297 (81.6)			
High-risk drinking, n (%)							194.50 (2)^a,b^	194.50 (2)^a,b^
Yes	723 (9.1)		223 (13.9)		358 (12.7)			
No	7187 (90.9)		1385 (86.1)		2457 (87.3)			
Walking, n (%)							7.20 (2)^a,c^	7.20 (2)^a,c^
Yes	3740 (47.3)		718 (44.7)		1325 (47.1)			
No	4170 (52.7)		890 (55.4)		1490 (52.9)			
Self-rated health, n (%)							683.35 (8)^a,c^	683.35 (8)^a,c^
Very good	246 (3.1)		88 (5.5)		76 (2.7)			
Good	2511 (31.7)		723 (45.0)		990 (35.2)			
Fair	3448 (43.6)		645 (40.1)		1369 (48.6)			
Poor	1590 (20.1)		147 (9.1)		361 (12.8)			
Very poor	115 (1.5)		5 (0.3)		19 (0.7)			
Number of chronic diseases, n (%)							3060.36 (4)^a,b^	
0	2739 (34.6)		1280 (79.6)		1146 (40.7)			
1	1764 (22.3)		267 (16.6)		816 (29.0)			
≥2	3407 (43.1)		61 (3.8)		853 (30.3)			
Disability, n (%)							223.74 (2)^a,b^	
Yes	594 (7.5)		27 (1.7)		137 (4.9)			
No	7316 (92.5)		1581 (98.3)		2678 (95.1)			
Hospital admission, n (%)							113.14 (2)^a,b^	
Yes	1142 (14.4)		146 (9.1)		332 (11.8)			
No	6768 (85.6)		1462 (90.9)		2483 (88.2)			
Usual source of care, n (%)							771.53 (2)^a,b^	
Yes	5015 (63.4)		639 (39.7)		1634 (58.1)			
No	2895 (36.6)		969 (60.3)		1181 (42.0)			
Health information seeking experience, n (%)							790.75 (2)^a,b^	
Yes	3768 (47.6)		1072 (66.7)		1644 (58.4)			
No	4142 (52.4)		536 (33.3)		1171 (41.6)			
HISB^d^ type^e^, n (%)							572.96 (2)^a,b^	
Active information-seeking category	2785 (73.9)		993 (92.7)		1285 (78.2)			
Passive information-acquisition category	982 (26.1)		78 (7.3)		359 (21.8)			
Monthly household income (1000 KRW)^g^, mean (SD)	2,323.41 (1,713.49)		2,976.28 (1,677.64)		2,764.25 (1,842.94)		528.33 (2, 7907)^b,f^	
Health-related quality of life (EQ-5D), mean (SD)	0.94 (0.10)		0.98 (0.05)		0.96 (0.07)		423.88 (2, 7907)^b,f^	
Number of outpatient visits, mean (SD)	19.81 (23.02)		10.10 (13.60)		15.93 (19.81)		411.89 (2, 7907)^b,f^	
Health literacy level, mean (SD)	10.96 (4.79)		14.10 (2.94)		12.43 (4.06)		1,337.46 (2, 7907)^b,f^	

a Chi-square test.

b *P*<.001.

c *P*<.05.

d HISB: health information–seeking behavior.

e Among participants who sought health information (n=3767), excluding 1 respondent who selected “other” as the only source.

f *F *test*.*

g KRW: Korean Won.

### Factors Associated With HISB Experience by Age Group

In the first stage of the primary analysis, a logistic regression analysis was used to examine factors associated with HISB experience. HL was significantly associated with HISB experience; participants with higher HL scores had higher odds of seeking health information (odds ratio [OR] 1.139, 95% CI 1.123‐1.154; *P*<.001). Other variables significantly associated with HISB experience included age, gender, marital status, education, income, private health insurance, current smoking status, health-related quality of life, and having a usual source of care. Compared with adults aged 65 years and older, those aged 19‐44 years had more than twice the odds of seeking health information (OR 2.072, 95% CI 1.690‐2.541), and those aged 45‐64 years also had higher odds (OR 1.728, 95% CI 1.502‐1.988). Conversely, men, individuals with lower education levels, current smokers, and those with higher EQ-5D scores had lower odds of seeking health information. Age-stratified logistic regression analyses showed different patterns of association across age groups. Among participants aged 19‐44 years, information seeking was more likely among women, married participants, those with two or more chronic conditions, and those with a usual source of care. Significant correlates of HISB experience in the 45‐64 age group included gender, education level, household income, current smoking, SRH, health-related quality of life, having a usual source of care, and HL. Among adults aged 65 years and older, education level, private health insurance, current smoking, number of outpatient visits, and HL were significantly associated with HISB experience (Table 3).

**Table 3.** Factors associated with health information–seeking experience by age group among Korean adults: a cross-sectional logistic regression analysis of the 2020‐2021 Korea Health Panel and 2021 Health Literacy Supplementary Survey.

Variables	Age group (years)			
	All (N=7910)	19‐44 (n=1608)	45‐64 (n=2815)	65+ (n=3487)
	OR^a^ (95% CI)	OR (95% CI)	OR (95% CI)	OR (95% CI)
Age (ref^b^=65+)				
19‐44	2.072^c^ (1.690‐2.541)	—^d^	—	—
45‐64	1.728^e^ (1.502‐1.988)	—	—	—
Sex (ref=female)				
Male	0.753^c^ (0.667‐0.850)	0.632^e^ (0.489‐0.816)	0.633^c^ (0.514‐0.778)	0.918 (0.759‐1.112)
Marital status (ref=not married)				
Married	1.655^c^ (1.364‐2.008)	1.798^f^ (1.422‐2.274)	0.877 (0.572‐1.346)	0.834 (0.357‐1.949)
Divorced, separated, or widowed	1.598^f^ (1.264‐2.019)	1.234 (0.618‐2.464)	1.085 (0.682‐1.727)	0.784 (0.334‐1.838)
Education level (ref=college or above)				
Elementary school or below	0.342^c^ (0.283‐0.412)	0.161 (0.015‐1.734)	0.245^c^ (0.174‐0.345)	0.448^c^ (0.330‐0.609)
Middle or high school	0.616 (0.540‐0.704)	0.676 (0.517‐0.885)	0.552 (0.456‐0.669)	0.656 (0.494‐0.871)
Monthly household income (in Korean won)	1.000^e^ (1.000‐1.001)	1.000 (0.999‐1.001)	1.001^f^ (1.000‐1.001)	1.001 (1.000‐1.001)
Health care coverage (ref=NHI)^g^				
Medical aid	0.838 (0.636‐1.104)	1.515 (0.608‐3.775)	0.615 (0.377‐1.005)	0.805 (0.549‐1.181)
Private health insurance (ref=no)				
Yes	1.217^e^ (1.063‐1.394)	1.522^f^ (1.002‐2.314)	1.004 (0.752‐1.341)	1.208^f^ (1.017‐1.435)
Economic activity (ref=no)				
Yes	0.948 (0.846‐1.063)	1.017 (0.790‐1.310)	0.945 (0.771‐1.158)	0.908 (0.764‐1.080)
Current smoking (ref=no)				
Yes	0.772^e^ (0.661‐0.902)	0.872 (0.639‐1.190)	0.775^f^ (0.612‐0.983)	0.652^e^ (0.485‐0.876)
High-risk drinking (ref=no)				
Yes	0.885 (0.741‐1.057)	0.935 (0.678‐1.289)	0.857 (0.664‐1.105)	0.915 (0.611‐1.372)
Walking (ref=no)				
Yes	1.054 (0.953‐1.166)	1.048 (0.840‐1.308)	1.082 (0.918‐1.274)	0.986 (0.837‐1.162)
Self-rated health (ref=very poor)				
Very good	0.961 (0.549‐1.683)	5.075 (0.597‐43.146)	0.436^f^ (0.140‐1.353)	0.965 (0.429‐2.175)
Good	1.045 (0.641‐1.706)	5.284 (0.652‐42.921)	0.435 (0.155‐1.218)	1.163 (0.623‐2.169)
Fair	1.370^e^ (0.847‐2.215)	6.106 (0.756‐49.293)	0.657 (0.237‐1.824)	1.296 (0.711‐2.362)
Poor	1.125 (0.702‐1.803)	6.717 (0.814‐55.429)	0.470 (0.168‐1.310)	1.117 (0.627‐1.991)
Number of chronic diseases (ref=0)				
1	1.097 (0.945‐1.274)	1.144 (0.831‐1.574)	0.954 (0.741‐1.178)	1.229 (0.889‐1.698)
≥2	1.044 (0.887‐1.228)	3.353^**^ (1.411‐7.972)	0.936 (0.741‐1.184)	1.212 (0.892‐1.646)
Health-related quality of life (EQ-5D)	0.303^e^ (0.162‐0.569)	0.155 (0.013‐1.857)	0.043^c^ (0.011‐0.160)	0.531 (0.235‐1.202)
Disability (ref =no)				
Yes	0.854 (0.691‐1.056)	0.423 (0.165‐1.083)	0.687 (0.455‐1.036)	0.941 (0.726‐1.219)
Hospital admission (ref =no)				
Yes	0.995 (0.858‐1.154)	0.943 (0.628‐1.416)	0.962 (0.741‐1.247)	0.997 (0.810‐1.226)
Number of outpatient visits	1.002 (1.000‐1.005)	1.007 (0.995‐1.020)	0.999 (0.994‐1.003)	1.004^f^ (1.000‐1.007)
Usual source of care (ref =no)				
Yes	1.518^c^ (1.347‐1.711)	1.819^c^ (1.426‐2.320)	1.579^c^ (1.311‐1.900)	1.214 (0.980‐1.503)
Health literacy	1.139^c^ (1.123‐1.154)	1.011 (0.972‐1.051)	1.138^c^ (1.113‐1.163)	1.178^c^ (1.154‐1.202)

a OR: odds ratio.

b ref: reference.

c *P*<.001.

d Not applicable.

e *P*<.01.

f *P*<.05.

g NHI: National Health Insurance.

### HL Scores by HISB Type and Information Source

**Figure 1.**
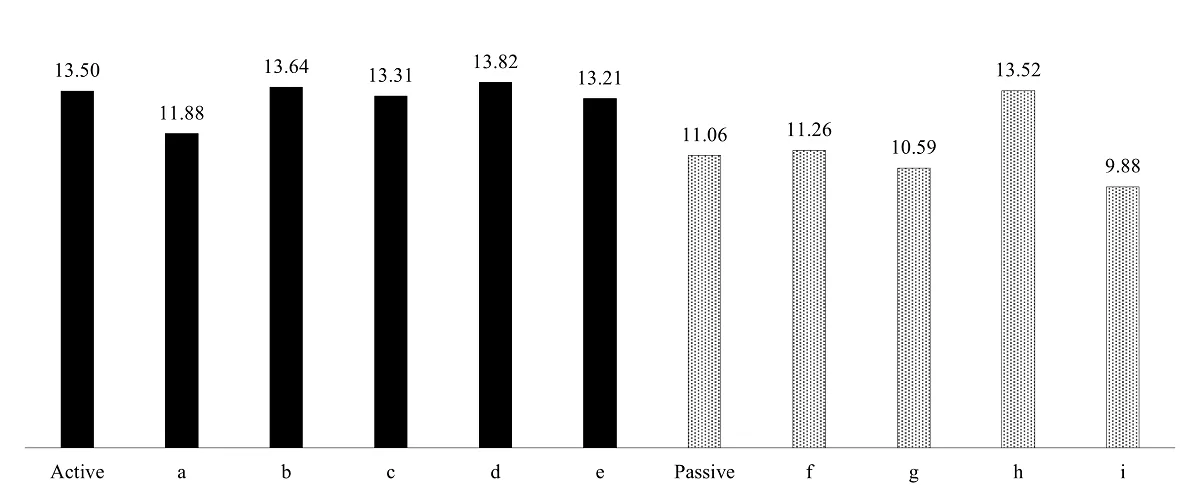
Health literacy scores by source-based health information–seeking behavior type and source among Korean adults: a cross-sectional analysis of the 2020‐2021 Korea Health Panel and 2021 health literacy supplementary survey. Active: active information seeking: (a) health care professionals (doctors, nurses, pharmacists, etc); (b) government or public sector channels (website, YouTube, and social network service); (c) hospital or public health channels (website, YouTube, social network service); (d) internet portals; e: YouTube (various sources). Passive: passive information acquisition: (f) television; (g) radio; (h) newspapers, magazines, or books (print and online); (i) family, friends, colleagues, and acquaintances.

**Figure 2.**
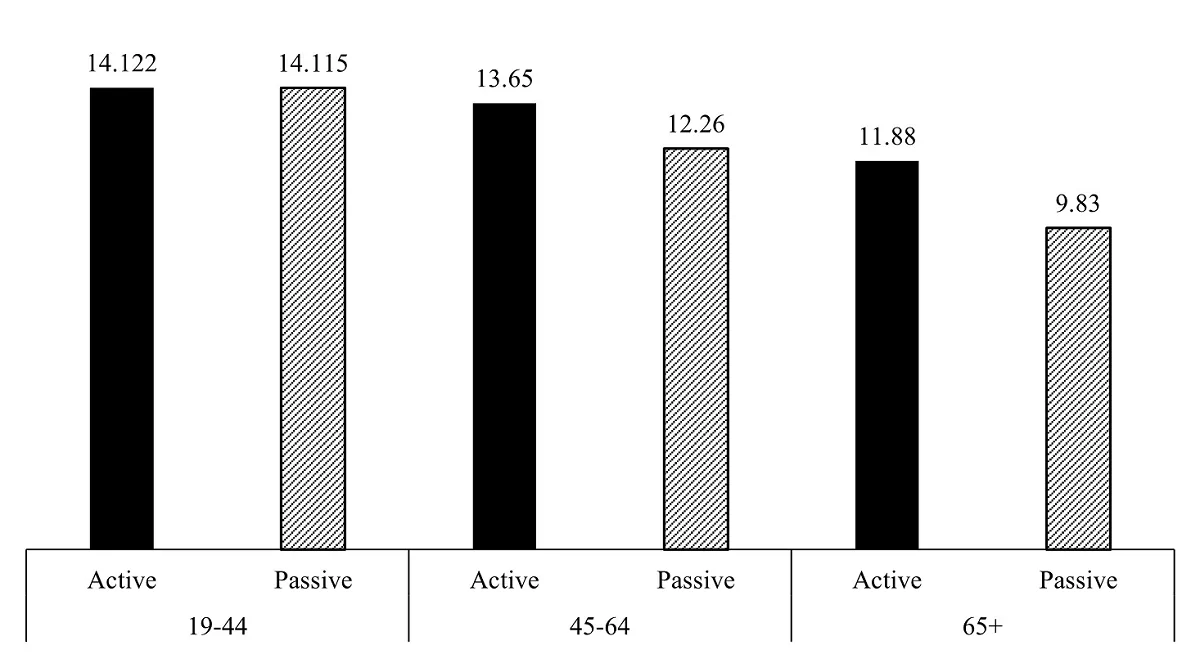
Health literacy scores by age group and source-based health information–seeking behavior type among Korean adults: a cross-sectional analysis of the 2020‐2021 Korea Health Panel and 2021 Health Literacy Supplementary Survey. Active: active information seeking; passive: passive information acquisition; age groups (years): 19‐44, 45‐64, and 65+.

Figures 1 and 2 illustrate differences in HL according to HISB type and information source. Overall, individuals classified in the active-seeking category had higher mean HL scores (mean 13.50, SD 3.18) than those in the passive acquisition category (mean 11.06, SD 4.17), with the highest mean score observed among users of internet portals (13.82, SD 2.94). Within the passive acquisition category, printed media users had the highest mean HL score (13.52, SD 2.99), whereas those relying on interpersonal sources (eg, family or acquaintances) had the lowest mean score (9.88, SD 4.26; Figure 1). Across age groups, mean HL scores were higher among individuals classified in the active-seeking category than among those in the passive acquisition category. However, the difference was smallest among adults aged 19‐44 years and was virtually absent in this group (14.122 vs 14.115; Figure 2).

### Factors Associated With HISB Type by Age Group

In the second stage of the primary analysis, logistic regression showed that participants with higher HL scores had higher odds of classification in the active-seeking category (OR 1.105, 95% CI 1.080-1.131; *P*<.001). Other significant correlates of HISB type included age, education level, SRH, number of chronic diseases, and health-related quality of life. Participants with higher education, fewer chronic diseases, and poorer SRH had higher odds of being classified as active-seeking. Compared with adults aged 65 years and older, individuals aged 19‐44 years had more than six times the odds of active-seeking classification (OR 6.072, 95% CI 4.374‐8.428), while those aged 45‐64 years had more than twice the odds (OR 2.582, 95% CI 2.096‐3.180). Age-stratified models showed further differences. In the youngest group, education level, SRH, and disability were significantly associated with HISB type, while HL was not. In this group, individuals with disabilities had higher odds of classification in the active-seeking category. Among adults aged 65 years and older, higher HL and higher EQ-5D scores were significant correlates of classification in the active-seeking category (Table 4).

**Table 4.** Factors associated with source-based health information–seeking behavior (HISB) type by age group among Korean adults: a cross-sectional logistic regression analysis of the 2020‐2021 Korea Health Panel and 2021 Health Literacy Supplementary Survey^a^.

Variables	Age group (years)			
	All (n=3767)	19‐44 (n=1071)	45‐64 (n=1644)	65+ (n=1052)
	OR^b^ (95% CI)	OR (95% CI)	OR (95% CI)	OR (95% CI)
Age group, years (ref^c^=65+)				
19‐44	6.072^f^ (4.374‐8.428)	—^g^	—	—
45‐64	2.582^d^ (2.096‐3.180)	—	—	—
Sex (ref=female)				
Male	1.017 (0.836‐1.238)	0.711 (0.410‐1.233)	0.807 (0.596‐1.092)	1.329 (0.980‐1.802)
Marital status (ref=unmarried)				
Married	0.973 (0.796‐1.189)	1.108 (0.659‐1.863)	0.802 (0.575‐1.119)	0.927 (0.675‐1.274)
Education level (ref=college or above)				
High school or below	0.639^f^ (0.520‐0.784)	0.555^f^ (0.320‐0.963)	0.592^e^ (0.447‐0.783)	0.757 (0.510‐1.122)
Monthly household income	1.000 (1.000‐1.001)	0.999 (0.998‐1.001)	1.000 (0.999‐1.001)	1.001 (1.000‐1.002)
Private health insurance (ref=no)				
Yes	1.025 (0.820‐1.280)	0.718 (0.231‐2.229)	1.008 (0.649‐1.568)	1.016 (0.767‐1.348)
Economic activity (ref=no)				
Yes	0.885 (0.736‐1.064)	0.921 (0.531‐1.599)	0.785 (0.584‐1.056)	0.969 (0.735‐1.277)
Current smoking (ref=no)				
Yes	0.952 (0.724‐1.252)	1.198 (0.593‐2.421)	1.116 (0.754‐1.650)	0.686 (0.419‐1.123)
High-risk drinking (ref=no)				
Yes	0.991 (0.729‐1.348)	0.596 (0.322‐1.105)	1.252 (0.819‐1.915)	0.982 (0.505‐1.907)
Walking (ref=no)				
Yes	1.065 (0.902‐1.257)	1.129 (0.698‐1.827)	0.881 (0.690‐1.126)	1.262 (0.968‐1.644)
Self-rated health (ref=poor)				
Good	0.921 (0.700‐1.211)	0.320^d^ (0.102‐0.999)	0.947 (0.608‐1.476)	0.972 (0.647‐1.460)
Fair	0.785^d^ (0.617‐0.999)	0.430 (0.142‐1.302)	0.757 (0.509‐1.124)	0.795 (0.564‐1.121)
Number of chronic diseases (ref=0)				
1	0.775 (0.609‐0.986)	1.318 (0.670‐2.595)	0.644 (0.467‐0.887)	0.962 (0.576‐1.605)
≥2	0.660^e^ (0.513‐0.850)	1.257 (0.393‐4.020)	0.551^e^ (0.392‐0.776)	0.821 (0.506‐1.332)
Health-related quality of life (EQ-5D; ref=<1)				
1	1.324^e^ (1.104‐1.589)	1.578 (0.878‐2.837)	1.196 (0.916‐1.562)	1.396^d^ (1.046‐1.865)
Disability (ref=no)				
Yes	0.812 (0.576‐1.144)	0.217^d^ (0.049‐0.961)	0.811 (0.437‐1.505)	0.877 (0.571‐1.348)
Hospital admission (ref=no)				
Yes	1.118 (0.879‐1.421)	1.292 (0.538‐3.100)	1.172 (0.789‐1.742)	1.043 (0.743‐1.465)
Number of outpatient visits	1.002 (0.998‐1.006)	0.990 (0.978‐1.003)	1.002 (0.994‐1.010)	1.004 (0.999‐1.009)
Usual source of care (ref=no)				
Yes	1.081 (0.888‐1.316)	0.862 (0.522‐1.425)	1.065 (0.806‐1.408)	1.293 (0.909‐1.840)
Health literacy	1.105^f^ (1.080‐1.131)	0.999 (0.915‐1.091)	1.111^f^ (1.073‐1.151)	1.122^f^ (1.084‐1.161)

a Active seeking was coded as 1 and passive acquisition as 0. HISB type was classified based on the respondent’s top-ranked information source.

b OR: odds ratio.

c ref: reference.

d *P*<.05.

e *P*<.01.

f *P*<.001.

g Not applicable.

### Sensitivity Analyses

In pooled multivariable logistic regression models including age×HL interaction terms, the joint test of the interaction terms was statistically significant for both HISB experience (Wald *χ*²_2_=57.84; *P*<.001) and HISB type (Wald *χ*²_2_=7.79; *P*=.02). Age-specific estimates showed that HL was significantly associated with HISB experience and classification in the active-seeking category among adults aged 45‐64 and ≥65 years, but not among those aged 19‐44 years. These findings were consistent with the age-stratified primary analyses (Table S1 in Multimedia Appendix 2).

In a separate sensitivity analysis, the Heckman-type 2-step analysis showed that respondents with higher HL scores had higher odds of classification in the active-seeking category in the primary conditional logistic model among those who engaged in HISB (OR 1.105, 95% CI 1.080‐1.130; *P*<.001). After the IMR was included in the second-stage logistic model, the IMR was statistically significant (OR 0.147, 95% CI 0.062‐0.348; *P*<.001), whereas the estimate for HL was attenuated and no longer statistically significant (OR 0.995, 95% CI 0.945‐1.048; *P*=.86). These findings indicate that the estimated association between HL and HISB type was sensitive to accounting for potential selection into the HISB type analytic sample (Table S2 in Multimedia Appendix 3).

## Discussion

### Principal Findings

This study demonstrated a distinct age-group pattern in HL, with higher mean HL scores among younger adults and lower mean scores among older adults. Given the cross-sectional design, this pattern should be interpreted as reflecting a combination of age and cohort differences rather than as evidence of HL decline caused by aging alone [36]. Differences in cognitive and sensory functions [16] and communication barriers in health care settings may partly contribute to the lower HL scores observed among older adults [24]. At the same time, cohort-specific differences in educational opportunities and formative exposure to digital technologies may also have contributed to distinct historical and technological environments [41]. These factors may also shape whether individuals engage in HISB and, among those who do, the source-based type of HISB observed in this study.

Table 5 provides an integrative summary of the main age-group patterns observed in this study. The matrix shows that the main correlates of HISB varied across age groups, with education level and health-related needs more salient among younger adults and HL emerging as a more prominent correlate among middle-aged and older adults, particularly for classification in the active-seeking category in the primary analyses. These contrasts may reflect both age-related differences in health needs and cohort-specific educational and information environments [36,37], consistent with recent evidence that information-source preferences differ across generations [41]. Viewed through the CMIS, these findings can be understood as age-group differences in the relative importance of individual antecedents, perceived health-related salience, and information-source factors rather than as a direct test of the full model [21,22]. In younger adults, education and perceived health needs may represent key antecedents and salience-related factors associated with information seeking, whereas HL may represent a more prominent cognitive and experiential resource among middle-aged and older adults, potentially supporting the recognition of information needs, evaluation of available channels, and engagement with health information (Table 5).

**Table 5.** Summary matrix of main correlates of health information–seeking behavior by age group among Korean adults: a cross-sectional analysis of the 2020‐2021 Korea Health Panel and 2021 Health Literacy Supplementary Survey.

Age group (years)	Observed HISB^a^ pattern	Main correlates	Interpretive summary
19‐44	Predominantly active seeking	Education level; health-related needs^b^	HISB was common and active, but HL^c^ was not significantly associated with HISB.
45‐64	Predominantly active seeking, with transitional features	HL; Education level; health-related needs^b^	HL was a significant correlate, alongside education and health-related needs^b^.
65+	Higher reliance on passive acquisition	HL; health-related quality of life	HL was significantly associated with classification in the active-seeking category.

a HISB: health information–seeking behavior.

b Health-related needs refer to health status indicators such as self-rated health, number of chronic diseases, disability status, or health-related quality of life, which were associated with HISB in different ways across age groups.

c HL: health literacy.

Building on these patterns, this analysis examined how HL, sociodemographic factors, and health-related factors were associated with the HISB experience and source-based HISB type across age groups. In the primary age-stratified analyses, HL was significantly associated with both HISB experience and classification in the active-seeking category among adults aged 45 years and older, but not among those aged 19‐44 years. The significant age ×HL interaction further supported heterogeneity in these associations across age groups. However, in the Heckman-type sensitivity analysis, the association between HL and HISB type was attenuated after accounting for potential selection into the HISB type analytic sample. Taken together, these findings suggest that HL may play a more prominent role in HISB among middle-aged and older adults, while the independent association between HL and HISB type should be interpreted cautiously because it was sensitive to the modeling of selection. This age-group pattern may reflect differences in cognitive processing needs, reliance on health care services, or familiarity with different information channels. The latter may also reflect cohort-specific exposure to changing media and digital environments [41]. In this context, HL may function not merely as a set of skills or knowledge but as a cognitive asset that facilitates engagement with, retention of, and application of health information [41,42]. This interpretation is consistent with a recent scoping review identifying eHealth literacy as an important individual resource for online HISB, but also showing that its role is embedded within broader educational, socioeconomic, health care, and digital-environmental conditions [43]. Prior evidence also points to a close relationship between HL and HISB, although its direction remains uncertain. Information seeking was more strongly associated with healthy lifestyle behaviors among individuals with higher HL [9], while older adults who regularly sought health content online had higher HL [10].

Among adults aged 19‐44 years, health information seeking was widespread, and more than 90% (993/1072) of seekers were classified in the active-seeking category. Despite this high engagement, HL was not significantly associated with either HISB experience or source-based HISB type in this group. Instead, the correlates differed by outcome: gender, marital status, multiple chronic conditions, and having a usual source of care were associated with HISB experience, whereas education level, SRH, and disability were associated with HISB type. While HL and education are often correlated, education, but not HL, was associated with classification in the active-seeking category, possibly reflecting broader educational resources, health awareness, or confidence in navigating information sources rather than specific information-processing skills [44]. Additionally, among those who engaged in HISB, individuals with better SRH had lower odds of classification in the active-seeking category, suggesting that perceived health-related need may be relevant to source-based information seeking [45,46]. These findings suggest that health-related needs and sociodemographic factors may be more salient correlates than HL among younger adults. However, because passive acquisition was uncommon in this age group, estimates for HISB type should be interpreted cautiously, and the nonsignificant association with HL should not be interpreted as evidence that HL is unimportant. A similar caveat applies to the predominance of classification in the active-seeking category among younger adults: this source-based classification does not directly capture intentionality, and frequent digital health information seeking does not necessarily translate into greater health empowerment or effective health management [47]. Therefore, interventions for this age group may benefit from emphasizing preventive care and future health risks while also strengthening the appraisal of digital health information and connections between digital information-seeking environments and professional guidance, including offline or telehealth-based support [47,48].

In the 45‐ to 64-year age group, the primary age-stratified analyses showed that HL and education were significantly associated with both HISB experience and source-based HISB type, whereas the number of chronic diseases was associated with HISB type. In the HISB type model, higher HL and education were associated with higher odds of classification in the active-seeking category [14,49], whereas a greater number of chronic diseases was associated with lower odds of such classification [2,50,51]. This finding contrasts with prior research and may reflect a gap between the need for information and the capacity to engage, potentially due to information overload, limited digital skills, or cognitive fatigue among individuals managing multiple health conditions [17,19]. These barriers are likely to be more pronounced among socioeconomically disadvantaged groups, where resource constraints can hinder the meaningful use of information [18]. In this context, HL may serve as an important resource for navigating information-access and processing challenges. Thus, strengthening HL, including digital HL, and ensuring access to accessible, trustworthy offline resources, such as community clinics and health centers, could support effective engagement with health information [52,53]. Tailored interventions are particularly relevant for middle-aged adults with limited incomes and lower education levels, who often face greater barriers to effective health communication [44].

Adults aged 65 years and older exhibited markedly lower levels of HISB, both in prevalence and in the proportion classified in the active-seeking category, compared with younger groups. In the primary age-stratified HISB type model, higher HL and EQ-5D scores were significantly associated with higher odds of classification in the active-seeking category. The association with EQ-5D suggests that overall functional health may be relevant to the capacity to engage with health information [2,51]. These findings indicate that HISB among older adults may be shaped not merely by perceived health needs, but also by the availability of cognitive and structural resources [14,50]. Consistent with the CMIS-informed interpretation introduced above, HL can be understood as an important resource for navigating cognitive, functional, and channel-related barriers to active engagement with health information [22,54]. At the same time, the higher proportion classified in the passive-acquisition category among older adults warrants attention, as prior media research has shown that health-related news may frame health problems in ways that emphasize individual responsibility over broader social determinants [55]. Such framing may be especially consequential for older adults with lower HL, who may have fewer opportunities or resources to verify, compare, or contextualize health information [54,56]. Therefore, interventions for older adults could combine efforts to strengthen HL with the reduction of access barriers through user-friendly technologies and community-based education tailored to their needs [53].

Taken together, these findings point to the value of strategies tailored to the needs and information environments of different age groups to support equitable and effective engagement with health information [48], consistent with recent evidence that health information source preferences differ across generations [41]. For older adults, who showed lower HISB prevalence and a higher proportion classified in the passive-acquisition category, interventions should not simply encourage more digital information seeking [54]. Rather, health systems could provide accessible proactive communication methods, such as short message service health alerts or automated reminders, while reducing digital barriers through user-friendly national health portals, community-based digital education, and peer-based technology mentoring programs [54,56]. These approaches may offer trusted entry points for older adults to gradually engage with digital health information while maintaining support from the community and health care professionals [48]. For younger adults, among whom most seekers were classified in the active-seeking category, the priority should shift from increasing information seeking itself to strengthening their ability to assess the credibility of, interpret, and apply the information they encounter [47]. Linking commonly used digital platforms with professional guidance, such as telehealth services or verified clinician-led information channels, may support more informed and effective use of health information [47,48]. As digital health environments increasingly include generative AI tools for health-related inquiries, tailored digital HL education may also be important for helping users critically evaluate AI-generated health information and reducing the risk of emerging informational inequalities across age groups [57].

### Limitations

This study has several limitations. First, because of its cross-sectional design, causal inferences cannot be drawn, and the temporal relationships between HL and HISB remain unclear. Because chronological age and birth cohort cannot be disentangled in cross-sectional data, the observed age-group pattern should be interpreted as potentially reflecting both age and cohort differences, rather than as evidence of physiological or cognitive changes attributable to aging alone [36]. For example, the lower prevalence of HISB and the higher proportion classified in the passive-acquisition category among older adults may partly reflect cohort differences in formative exposure to the internet and digital technologies, rather than aging alone [47].

Second, the analysis was limited to respondents who participated in the HL supplement of the KHP and had complete data for the variables included in the analyses, a subsample of the broader national cohort. As appropriate weights specifically constructed for this restricted analytic subsample were not available, the analyses were conducted without applying survey weights. Therefore, the reported estimates should be interpreted as unweighted associations within the analytic sample rather than as nationally representative population estimates, and their generalizability may be limited.

Third, unmeasured confounding may persist despite adjustment for several socioeconomic and baseline health status variables. In particular, the KHP dataset did not include detailed measures of physical access to digital devices, internet affordability, or perceived accessibility and usability of online health information channels, all of which may influence both HL and HISB, particularly among older adults and other groups with limited digital resources [22,47,54,56]. Other potentially relevant factors, such as digital self-efficacy, prior experience with online health services, trust in specific information sources, and cognitive functioning, were also not directly measured. A recent scoping review among individuals with diabetes identified multilevel determinants of online HISB spanning individual, interpersonal, health care, and digital-environmental domains, further underscoring the possibility that relevant contextual factors were not fully captured in the present analysis [43].

Fourth, HISB type was operationalized as a source-based proxy using the respondent’s highest-ranked information source. This approach did not directly assess intentionality and could not capture the simultaneous use of multiple sources, changes in information-seeking mode across situations, or variation in how actively individuals engaged with a given source. This binary classification into active seeking and passive acquisition may not fully capture the complexity of real-world HISB.

Fifth, the HISB type analysis was conditional on respondents having engaged in HISB, creating the possibility of selection into the analytic sample. In the Heckman-type sensitivity analysis, the estimated association between HL and HISB type was attenuated after the IMR was included. Accordingly, the primary findings regarding HL and HISB type should be interpreted with caution because they were sensitive to modeling potential selection.

Sixth, passive acquisition was uncommon among adults aged 19‐44 years. The relatively small number of observations in this outcome category may have reduced the precision and stability of estimates in the youngest age group. Consequently, the nonsignificant association between HL and HISB type in this group should not be interpreted as evidence that no association exists.

Seventh, although HL was treated as a focal independent variable in this study, its role may be more nuanced, potentially functioning as a mediator or moderator in broader health engagement pathways. The analyses also could not evaluate reciprocal relationships in which engagement with health information may itself contribute to HL over time. Additionally, the assumption that classification in the active-seeking category is inherently beneficial warrants caution, as active engagement with health information may sometimes lead to information overload or exposure to misinformation [52].

Eighth, the dataset did not allow us to account for potentially important age-varying factors, including eHealth literacy and trust in specific media or information sources, that may shape the association between HL and HISB. Among younger adults, the ability and confidence to navigate online platforms may be more salient than general HL, whereas older adults’ greater reliance on passive acquisition may partly reflect trust in familiar sources such as television or interpersonal networks [29,41]. Older adults’ engagement with digital health services may also be constrained by technology-related anxiety, privacy concerns, and concerns that digitally mediated care could weaken interpersonal connections, which may reinforce a preference for in-person care [58]. The analyses did not capture patient activation or discussions of online health information with health care providers, which may influence whether information seeking translates into patient-centered communication [59]. Future studies should incorporate these digital, psychological, and interpersonal factors to clarify the mechanisms underlying age-group differences in HL and HISB. Finally, data were collected during the COVID-19 pandemic, and changes in health service accessibility and communication patterns may have influenced HL and information-seeking behaviors.

### Conclusions

This study identified clear age-group patterns in the association between HL and HISB among Korean adults, underscoring that the role of HL and source-based HISB type differed across age groups. In the primary analyses, higher HL was associated with HISB experience and classification in the active-seeking category among middle-aged and older adults, whereas educational attainment and health-related needs were more salient among younger adults. However, because the association between HL and HISB type was sensitive to accounting for potential selection into the HISB type analytic sample, this specific association should be interpreted cautiously. At the same time, the broader age-group patterns observed across HISB experience and source-based HISB type highlight the value of designing strategies tailored to the needs and information environments of different age groups to improve access to and effective use of health information. Rather than focusing solely on strengthening HL, future interventions should also consider structural, motivational, and digital access factors to promote equitable engagement with health information across age groups.

## Supplementary material

10.2196/86992 Multimedia Appendix 1 Flow diagram of sample selection among Korean adults.

10.2196/86992 Multimedia Appendix 2 Sensitivity analysis of age×health literacy interactions for health information–seeking experience and behavior type among Korean adults.

10.2196/86992 Multimedia Appendix 3 Sensitivity analysis comparing logistic models of HISB type with and without the inverse Mills ratio among Korean adults who engaged in HISB. HISB: health information–seeking behavior.

## Abbreviations

CMIS: Comprehensive Model of Information Seeking
HISB: health information–seeking behavior
HL: health literacy
HLS-EU-Q16: European Health Literacy Survey Questionnaire-16-item
IMR: inverse Mills ratio
KHP: Korea Health Panel
OR: odds ratio
SAS: Statistical Analysis System
SRH: self-rated health
