# Supplementary material for: Age-Related Patterns in Health Literacy and Health Information–Seeking Behavior in a Korean Population: Cross-Sectional Study
Source: JMIR Public Health Surveill. 2026 Sep 23;12:e86992. doi: 10.2196/86992 (PMC13600592; doi:10.2196/86992)
Supplement: Supplementary file 1 [file publichealth-v12-e86992-s001.docx]

 **Figure S1**. Flow diagram of sample selection among Korean adults: a cross-sectional analysis of the 2020–2021 Korea Health Panel and 2021 Health Literacy Supplementary Survey
