# Supplementary material for: Age-Related Patterns in Health Literacy and Health Information–Seeking Behavior in a Korean Population: Cross-Sectional Study
Source: JMIR Public Health Surveill. 2026 Sep 23;12:e86992. doi: 10.2196/86992 (PMC13600592; doi:10.2196/86992)
Supplement: Supplementary file 2 [file publichealth-v12-e86992-s002.docx]

**Table S1**. Sensitivity analysis of Age × Health Literacy interactions for health information-seeking experience and behavior type among Korean adults: a cross-sectional analysis of the 2020-2021 Korea Health Panel and 2021 Health Literacy Supplementary Survey

| Variable | Model 1: HISB Experience | Model 2: Active vs. Passive HISB |
| --- | --- | --- |
| Joint interaction test for Age × HL | Wald χ² = 57.84, *p* < 0.001 | Wald χ² = 7.79, *p* = 0.020 |
| **Age-specific effects of HL** | **OR (95% CI)** | **OR (95% CI)** |
| 19–44 | 1.006 (0.970-1.044) | 0.989 (0.909–1.076) |
| 45–64 | 1.138 (1.114–1.162)^***^ | 1.110 (1.072–1.148)^***^ |
| ≥ 65 | 1.180 (1.157–1.204)^***^ | 1.125 (1.088–1.163)^***^ |

Models were adjusted for the same sociodemographic, health behavior, health status, and healthcare utilization covariates used in the main multivariable analyses. The joint interaction test assessed the overall statistical significance of the Age × HL interaction terms in the pooled models. Age-specific ORs represent the adjusted odds ratios for each one-point increase in HL within each age group, derived from the pooled interaction models. In Model 2, active seeking was coded as 1 and passive acquisition as 0.

*** *p* < 0.001

CI, confidence interval; HISB, health information-seeking behavior; HL, health literacy; OR, odds ratio;
