# Supplementary material for: Age-Related Patterns in Health Literacy and Health Information–Seeking Behavior in a Korean Population: Cross-Sectional Study
Source: JMIR Public Health Surveill. 2026 Sep 23;12:e86992. doi: 10.2196/86992 (PMC13600592; doi:10.2196/86992)
Supplement: Supplementary file 3 [file publichealth-v12-e86992-s003.docx]

**Table S1**. Sensitivity analysis comparing logistic models of HISB type with and without the inverse Mills ratio among Korean adults who engaged in HISB: a cross-sectional analysis of the 2020–2021 Korea Health Panel and 2021 Health Literacy Supplementary Survey

| Variable | Primary HISB Type Logistic Model without IMR  OR (95% CI) | Heckman-Type Second-Stage Logistic Model with IMR  OR (95% CI) |
| --- | --- | --- |
| **Health literacy, per 1-point increase** | 1.105 (1.080-1.130)^***^ | 0.995 (0.945-1.048) |
| **Age groups, years** (ref = 65+) |  |  |
| 19–44 | 6.072 (4.374-8.428)^***^ | 3.697 (2.491-5.485)^***^ |
| 45–64 | 2.582 (2.096-3.180)^***^ | 1.628 (1.218-2.177)^***^ |
| **Number of chronic diseases** (ref = 0) |  |  |
| 1 | 0.775 (0.609-0.986)^*^ | 0.725 (0.568-0.924)^**^ |
| ≥ 2 | 0.660 (0.513-0.850)^**^ | 0.642 (0.499-0.828)^***^ |
| EQ-5D index, 1 vs. < 1 | 1.324 (1.104-1.589)^**^ | 1.452 (1.204-1.750)^***^ |
| Inverse Mills ratio | - | 0.147 (0.062-0.348)^***^ |

Selected estimates are presented. Both models were estimated among respondents who reported HISB experience (n = 3,767), with active seeking coded as 1 and passive acquisition as 0. The Heckman-type second-stage model retained the same covariate coding and adjustment set as the primary HISB type model and additionally included the IMR, which was derived from a first-stage probit model estimating selection into the HISB type analytic sample. Both models were adjusted for the same sociodemographic, health behavior, health status, and healthcare utilization covariates used in the primary HISB type analysis. ORs for HL represent the association with a 1-point increase in HL. Analyses were unweighted. An em dash indicates the reference category or that the variable was not included in the model.

* *p* < 0.05; ** *p* < 0.01; *** *p* < 0.001

CI, confidence interval; EQ-5D, EuroQol 5-Dimension index; HISB, health information-seeking behavior; HL, health literacy; IMR, inverse Mills ratio; OR, odds ratio
